# Pulmonary Capillary Wedge Pressure during Exercise Is Prognostic for Long-Term Survival in Patients with Symptomatic Heart Failure †

**DOI:** 10.3390/jcm11195901

**Published:** 2022-10-06

**Authors:** Christoph Ahlgrim, Sascha Kocher, Jan Minners, Nikolaus Jander, Gianluigi Savarese, Franz-Josef Neumann, Thomas Arentz, Amir Jadidi, Björn Mueller-Edenborn

**Affiliations:** 1 Department of Cardiology and Angiology II, Heart Center, University of Freiburg, 79106 Freiburg, Germany; 2 Division of Cardiology, Department of Medicine, Karolinska Institutet, Solna, 171 64 Stockholm, Sweden

**Keywords:** exercise hemodynamics, heart failure, right heart catheterization, exercise testing

## Abstract

Aims: Exercise stress testing can stratify specific populations of heart failure patients for mortality risk, but is not universally applied. The aim of the present study was to investigate the prognostic capabilities of invasive exercise testing in a real-world cohort of suspected heart failure patients in whom non-cardiac causes of dyspnea were excluded. Methods: We retrospectively analyzed the survival of 682 patients who underwent right heart catheterization at rest and during exercise between 2007 and 2017 for dyspnea and expected heart failure. Pulmonary capillary wedge pressure (PCWP) at rest and the PCWP response to exercise, expressed as the ratio of PCWP at peak exercise to workload normalized to body weight (PCWL (mmHg/W/kg)), were determined. Mortality data were retrieved from the official German death registry. Results: Over a median follow-up period of 8.5 years, PCWL is a stronger predictor of all-cause mortality than PCWP. Patients featuring a reduced left ventricular ejection fraction (LVEF; <50%), but favorable response to exercise (PCWL <34.7 mmHg/W/kg), have a similar mortality risk to patients with a normal LVEF and low PCWL (hazard ratio (HR) 1.180, 95% CI 0.48–2.91, *p* = 0.719). Irrespective of LVEF, an increased PCWL during exercise was associated with a significantly increased mortality (HR 1.950 with preserved LVEF, 95% CI 1.12–3.34, *p* = 0.018; and HR 3.212 with impaired LVEF, 95% CI 1.75–5.70, *p* < 0.001). Conclusions: In patients with clinical heart failure, invasive exercise testing improves the prediction of mortality. Subjects with a favorable response to exercise have a relatively low mortality irrespective of left ventricular systolic function.

## 1. Introduction

Heart failure is a syndrome that is defined by clinical signs and symptoms related to cardiac dysfunction. Historically, patients with heart failure are divided into subtypes based on left ventricular systolic function: those with heart failure and preserved left ventricular ejection fraction (HFpEF) and patients with heart failure with reduced LVEF ≤40% (HFrEF) [1]. More recently, patients with an LVEF between 40% and 49% were considered as a distinctive group named heart failure with mildly reduced ejection fraction (HFmrEF) [2].

As all types of heart failure are associated with substantial long-term mortality [3,4,5], the estimation of an individual’s risk for death is an important part of patient-centered care. Various clinical or diagnostic markers were investigated in specific heart failure subtypes to improve risk stratification, but their clinical applicability is relatively limited. In particular, left ventricular systolic function proved to be a less important predictor of death than previously anticipated, with significant morbidity and mortality reported also in patients with preserved systolic function [4].

A key feature of all subtypes of advanced heart failure is the clinical inability to adapt to exercise, resulting in dyspnea under exertion at various stages of intensity. Failure to adapt to exercise was previously related to mortality in studies investigating specific subtypes of heart failure such as HFpEF [6] or end-stage HFrEF [7], but it is unclear whether these findings can be extrapolated to the heterogeneous cohort of clinical heart failure syndrome.

Another possible limitation in many studies on exercise tolerance is patient cooperation, with premature abortion of exercise being an important source of error. Compared to pulmonary capillary wedge pressure (PCWP) at peak exercise as often used, PCWL, calculated as PCWP at peak exercise to workload normalized to body weight, was demonstrated to be less dependent on patient cooperation and was validated previously in heart failure patients [6,8].

The aim of the current study was to evaluate the performance of invasive exercise testing including PCWL to predict death in a large real-world cohort of patients with symptomatic heart failure.

## 2. Methods

### 2.1. Study Population

In this retrospective study, consecutive patients referred to our center for unexplained dyspnea of NHYA-classes II and III and undergoing right heart catheter between January 2007 and December 2017 were screened for inclusion. Patients were considered eligible when non-cardiac causes of dyspnea such as abnormal pulmonary function and anemia were excluded and right heart catheterization was considered the next appropriate step in the diagnostic workup. Patients with prior cardiac surgery including pacemaker implantation or acute myocardial infarction in the preceding three months were excluded from further analysis. All patients had their history taken and were clinically examined, and echocardiography was performed to determine cardiac dimensions and left ventricular systolic function. In the context of this study, medications that are part of optimal medical therapy for heart failure at baseline were considered in patients who took the respective medication for four weeks or more preceding the index hospitalization.

Follow-up for mortality was performed through the official German death registry. For 5/687 patients (0.7%), mortality data were unavailable in these records because of the relocation of the patients to a place outside of Germany. These patients were excluded from further analysis. The date of right-heart catheterization was defined as the index date and beginning of follow-up. The primary endpoint was the performance of variables quantifying the cardiac response to invasive exercise testing to predict all-cause mortality. The study was approved by the institutional review board of the University of Freiburg.

### 2.2. Right Heart Catheterization and Exercise Testing

Right heart catheterization was performed as described previously by our group in detail [6]. Briefly, resting pressures were measured in the supine position using a Swan–Ganz catheter inserted via the brachial vein. Following resting measurements and estimation of PCWP, all patients performed cycle ergometry starting at 25 W or 50 W based on individual physical capacity, with increments of 25 W or 50 W until exhaustion. Every exercise level was maintained for 5 min. During exercise, the response of pulmonary capillary wedge pressure to exercise was measured and expressed as PCWL.

### 2.3. Statistical Analysis

PCWP at rest and PCWL during exercise were analyzed as continuous and dichotomized variables. Study subjects were stratified into heart failure subtypes based on LVEF (LVEF ≥50% in HFpEF, LVEF 40–49% in HFmrEF, and LVEF <40% in HFrEF).

Continuous variables are reported as mean and standard deviation and were compared using Student’s t-test after confirming normality using the Kolmogorov–Smirnov test. Intergroup comparisons for non-normally distributed PCWP and PCWL were performed using Mann–Whitney U test. Categorical variables are reported as percentages, and intergroup comparisons were performed using the chi^2^ or Fisher’s exact test as appropriate. Survival curves were expressed using the Kaplan–Meier methods. Hazard ratios were calculated using Cox regression analysis.

For multivariate Cox regression models, clinical and echocardiographic variables that predicted mortality in univariate analysis at an alpha-error of <0.05 (see Table 1) were included in addition to PCWP and/or PCWL. Receiver operating characteristic (ROC) curves were constructed to assess c-statistics of pertinent variables for mortality. Optimal cutoff points were identified by the Youden index. ROC curves were compared using the previously reported method by DeLong et al. using the pROC package in R. The individual contribution of a parameter to the regression models, the integrated discrimination index (IDI) [9], was calculated using the survIDINRI package. All other statistical analyses were performed using SPSS V25.0 (IBM, Ehningen, Germany). An alpha level of 0.05 was accepted for statistical significance.

## 3. Results

### 3.1. Patient Characteristics

A total of 687 patients with suspected symptomatic heart failure that underwent right heart catheterization between January 2007 and December 2017 were included in this study. Mortality data were available for 682/687 patients (99.3%). These patients constitute the study sample. Median follow-up was 8.5 years (IQR 5.2–10.8 years). A total number of 231 patients (33.6%) died during follow-up (Table 1). Resting and exercise hemodynamics in survivors and non-survivors are described in Table 2.

### 3.2. Pulmonary Capillary Wedge Pressure and Survival

**Figure 1:**
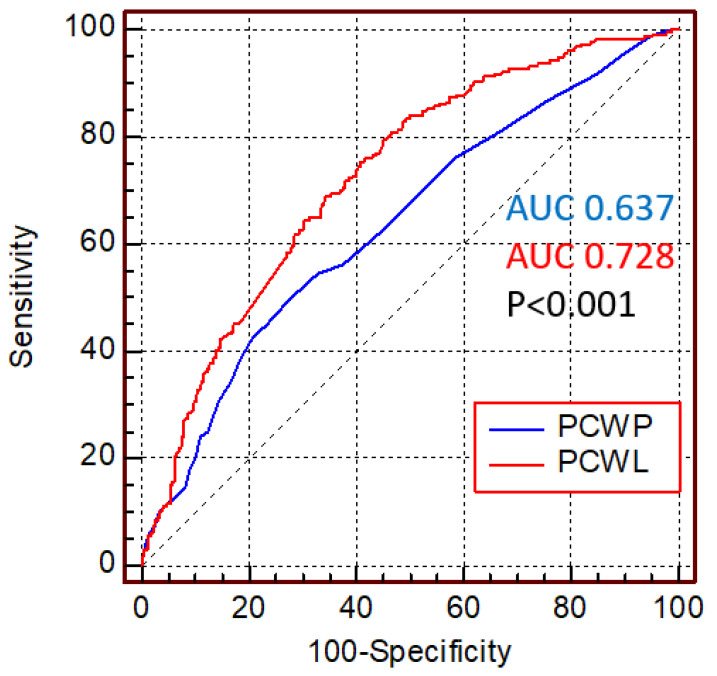
Diagnostic properties of pulmonary capillary wedge pressures at rest and during exercise to predict survival. Receiver operating curve analysis comparing the diagnostic properties of pulmonary capillary wedge pressure at rest (PCWP, in blue) and pulmonary capillary wedge pressure during exercise, expressed as peak exercise to workload normalized to body weight (PCWL, in red). ROC curves were compared using the previously reported method by DeLong et al.

**Figure 2:**
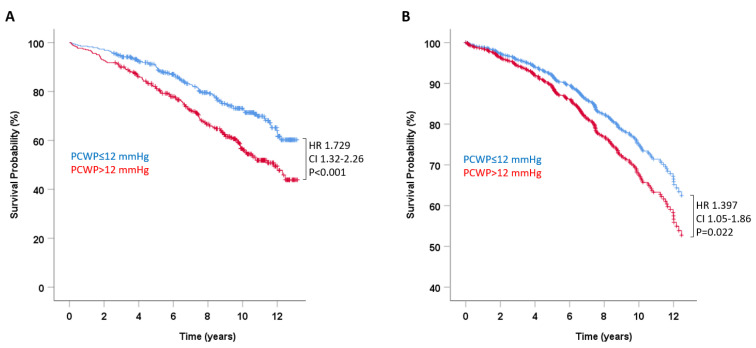
Pulmonary capillary wedge pressures at rest and survival in heart failure. Survival probability in patients with normal (≤12 mmHg) and elevated (>12 mmHg) pulmonary capillary wedge pressure (PCWP) at rest unadjusted (**A**) and adjusted for clinical and echocardiographic variables that predicted mortality in univariate analysis (see Table 1) (**B**).

Pulmonary capillary wedge pressure (PCWP) at rest was significantly associated with all-cause mortality. In univariate analysis, the hazard ratio for death per millimeter mercury increase in PCWP was 1.048 (95% confidence interval (CI) 1.032–1.064, *p* < 0.001). ROC analysis for death yielded an AUC of PCWP at rest of 0.637 (95% CI 0.59–0.68; Figure 1). Dichotomizing PCWP for the previously established criterion for left atrial hypertension and HFpEF of 12 mmHg (341 of 687 patients had PCWP > 12 mmHg, 49.6%) yielded an HR for death in univariate analysis of 1.729 (95% CI 1.32–2.26, *p* < 0.001; Figure 2A).

The association of PCWP with mortality persisted after multivariate adjustment to significant baseline variables outlined in Table 1. This was true both for PCWP as a continuous variable (HR 1.039, 95% CI 1.02–1.06, *p* < 0.001) and for PCWP dichotomized for left atrial pressure of >12 mmHg (HR 1.397, 95% CI 1.05–1.86, *p* = 0.022; Figure 2B).

**Figure 3:**
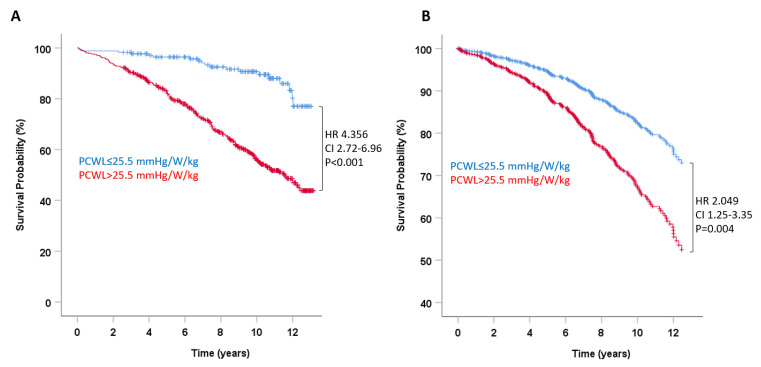
Pulmonary capillary wedge pressure during exercise and survival in heart failure. Survival probability in patients with normal (≤25.5 mmHg/W/kg) and pathologic (>25.5 mmHg/W/kg) response to exercise, expressed as peak exercise to workload normalized to body weight. (**A**) Unadjusted survival probability; (**B**) survival probability adjusted for clinical and echocardiographic variables that predicted mortality in univariate analysis (see Table 1).

The hemodynamic response to exercise quantified as PCWL was also significantly associated with mortality. The crude HR for death was 1.028 (95% CI 1.02–1.04, *p* < 0.001) per 10 mmHg/W/kg rise in PCWL. In ROC analysis, the AUC for PCWL to predict death was 0.728 (95% CI 0.689–0.767; Figure 1), representing a significant increase as compared to PCWP (*p* < 0.001). In line with these findings, adding PCWL to PCWP improved the prediction of survival using IDI reclassification (0.01, *p* = 0.04). Dichotomizing PCWL for the previously established cutoff value for a pathologic response to exercise in patients with HFpEF [6] of >25.5 mmHg/W/kg yielded an HR for death of 4.356 (95% CI 2.72–6.96, *p* < 0.001, Figure 3A).

The prognostic value of PCWL prevailed after adjustment for significantly different pertinent baseline values, both as a continuous variable (HR per 10 mmHg/W/kg increment 1.017, 95% CI 1.01–1.03, *p* = 0.005) and dichotomized for a cutoff value of 25.5 mmHg/W/kg (HR 2.049, 95% CI 1.25–3.35, *p* = 0.004; Figure 3B).

### 3.3. Pulmonary Capillary Wedge Pressure during Exercise and Survival in Heart Failure Stratified by Left Ventricular Systolic Function

Study patients were stratified according to their echocardiographic left ventricular systolic function. A total of 514 patients (75.4%) had preserved systolic function with LVEF ≥50% (HFpEF), and 168 patients (24.6%) had impaired LVEF < 50%: 72 patients (10.6%) with heart failure with mid-range ejection fraction (HFmrEF; LVEF 40–49%) and 96 patients (14.1%) with heart failure with reduced ejection fraction (HFrEF; LVEF < 40%). Thirty-eight percent of patients with HFrEF had ischemic cardiomyopathy, with the remaining suffering from dilated and/or structural cardiomyopathy.

LVEF as a continuous variable was not associated with all-cause mortality (HR 0.995; 95% CI 0.99–1.00, *p* = 0.206). In addition, dichotomizing patients into preserved LVEF ≥ 50% vs. impaired LVEF < 50% did not reach statistical significance for prediction of death (HR 1.01, 95% CI 0.82–1.478, *p* = 0.525). Table 3 illustrates the characteristics of the study group stratified to LVEF (LVEF ≥ 50% vs. <50%).

In patients with preserved LVEF (*n* = 514), the HR for death was 1.030 (95% CI 1.02–1.04, *p* < 0.001) per 10 mm/W/kg increment in PCWL and 4.48 (95% CI 2.64–7.61, *p* < 0.001) when PCWL was dichotomized for the previously established cutoff value for HFpEF of 25.5 mmHg/W/kg [6].

In patients with impaired EF (LVEF <50%), PCWL as a continuous variable was equally associated with survival (HR 1.022, 95% CI 1.01–1.04, *p* = 0.016). Compared to patients with preserved LVEF, patients with impaired LVEF had higher pulmonary capillary wedge pressures during exercise (56.6 (31.9–66.8) mmHg/W/kg vs. 40.5 (23.6–56.6) mmHg/W/kg, *p* < 0.001 for PCWL; Figure S1). As a result, ROC analysis of PCWL for mortality in patients with impaired LVEF resulted in a higher cutoff value for PCWL of 34.7 mmHg/W/kg. Dichotomizing PCWL for this cutoff value in patients with impaired LVEF yielded an HR for death of 3.630 (95% CI 1.65–8.0, *p* < 0.001) that persisted in multivariate analysis (HR 2.637, 95% CI 1.18–5.88, *p* = 0.018). In contrast, applying the cutoff value of 25.5 mmHg/W/kg as previously demonstrated for preserved LVEF failed to predict death in this population (HR 1.969, 95% CI 0.70–5.55, *p* = 0.20).

**Figure 4:**
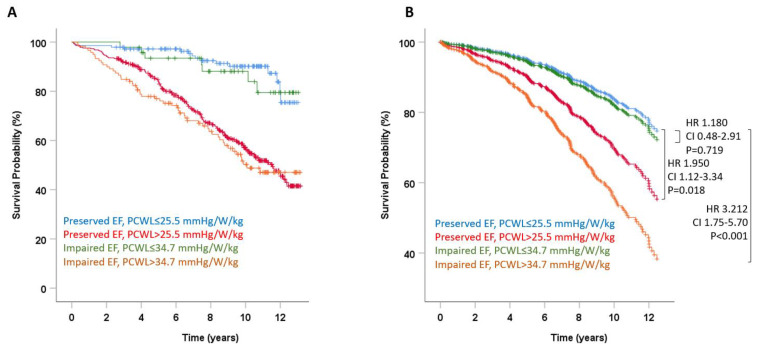
Pulmonary capillary wedge pressure (adjusted for left ventricular ejection fraction) during exercise and survival in heart failure. Survival probability in patients with clinical heart failure expressed as peak exercise to workload normalized to body weight. The cutoff for unfavorable response to exercise was independently determined and validated for patients with preserved left ventricular ejection fraction (EF ≥ 50%; PCWL unfavorable when >25.5 mmHg/W/kg) and impaired EF (<50%; PCWL unfavorable when >34.7 mmHg/W/kg). (**A**) Unadjusted survival probability; (**B**) survival probability adjusted for clinical and echocardiographic variables that predicted mortality in univariate analysis (see Table 1).

The study population was next stratified for systolic function and using LVEF-specific cutoffs quantifying the response to exercise: preserved LVEF with normal and pathologic response to exercise (LVEF ≥ 50%/PCWL < 25.5 mmHg/W/kg, *n* = 141; LVEF ≥ 50%/PCWL ≥ 25.5 mmHg, *n* = 374), and impaired LVEF with normal and pathologic response to exercise (LVEF < 50%/PCWL < 34.7 mmHg/W/kg, *n* = 46; LVEF < 50%/PCWL ≥ 34.7 mmHg/W/kg, *n* = 121). Considering patients with preserved LVEF and normal response to exercise as a reference group (LVEF ≥ 50%/PCWL < 25.5 mmHg/W/kg), an increase in PCWL above the LVEF-specific cutoff (>25.5 mmHg/W/kg for preserved LVEF or >34.7 mmHg/W/kg for impaired LVEF) was independently associated with mortality in unadjusted and adjusted models (Figure 4A,B). Depending on the systolic function, these patients carried a 1.95-fold (with preserved LVEF, 95% CI 1.12–3.34, *p* = 0.018) to 3.2-fold (with impaired LVEF, 95% CI 1.75–5.70, *p* < 0.001) increased risk of death (Figure 4B). Patients with impaired LVEF but featuring a response to exercise below the cutoff of 34.7 mmHg/W/kg had a risk of death comparable to the reference group (HR 1.180, 95% CI 0.48–2.91, *p* = 0.719).

## 4. Discussion

We report two main findings on the prognostic utility of exercise hemodynamics in heart failure: First, invasive quantification of the hemodynamic response to exercise in symptomatic heart failure improves risk stratification for death in adjunction to the qualitative systolic left ventricular function (preserved (LVEF>50%) vs. impaired (LVEF<50%)). Second, it allows the identification of an important group of patients with impaired systolic function but hemodynamic tolerance to exercise and relatively low risk of death that is comparable to control patients.

### 4.1. Hemodynamics of Heart Failure

Increased intracardiac pressures and reduced cardiac output at rest or during exercise are the hallmarks of overt heart failure. Exercise testing is, however, not part of the diagnostic routine in most patients with suspected heart failure. As a result, insights gained from exercise testing are mostly derived from very specific subtypes of heart failure, such as end-stage heart failure [10] or heart failure with preserved ejection fraction (HFpEF) [6,11]. If used in the clinical workup of heart failure patients, exercise testing is currently mostly performed using spiroergometry, which allows the quantification of functional capacity by indirect estimation of exercise hemodynamics such as VO2peak (maximum oxygen uptake) or VE-VCO2-slope (respiratory efficiency).

However, the functional capacity of heart failure patients can be impaired at various levels such as lung diffusing capacity [12] or peripheral oxygen extraction [13] that are not necessarily related to cardiac output or load tolerance. Invasive exercise testing in contrast directly quantifies the intracardiac pressure response during exercise, limiting potential confounding factors. Our data demonstrate that the load characteristics obtained invasively, particularly during threshold exercise, are very strong predictors of mortality in symptomatic heart failure irrespective of systolic function. These findings are supported by previous studies from our group and others who demonstrated the benefit of exercise testing for risk stratification in HFpEF using PCWL or PCWP indexed to cardiac output (expressed as PCWP/CO slope) [6,11]. Therefore, it appears reasonable that when the decision for right heart catheterization is made, the exam should be complemented with stress testing to assess cardiac pressures under load.

To our knowledge, the cohort investigated in this study is the largest study sample of heart failure patients of any type with measurements of invasive exercise hemodynamics and, for the first time, allows quantification of the mortality risk that can directly be attributed to load intolerance of the heart.

### 4.2. Left Ventricular Function and Death in Heart Failure

Despite its limitations, quantification of LVEF is still the cornerstone for directing therapy and estimating prognosis in chronic heart failure [14]. The prognostic value of LVEF is, however, driven by patients with advanced left ventricular systolic dysfunction (LVEF < 40%), in whom declines in LVEF are indeed associated with increased mortality rates [15].

This association is less obvious in the large group of patients with mid-range systolic dysfunction, as in HFmrEF (LVEF 40% to 50%), and in patients with preserved systolic function (HFpEF)^15^. Our current study features a real-world cohort encompassing all these clinical heart failure subtypes. In contrast to LVEF, invasive hemodynamic testing ubiquitously identifies study subjects with a low mortality risk in all these heart failure types, as indicated by a normal response to exercise, i.e., PCWL values below the cutoff values for preserved and impaired LVEF (<25.5 mmHg/W/kg for preserved LVEF and <34.7 mmHg/W/kg for impaired LVEF). These findings emphasize that exercise hemodynamics and the clinical response to exercise, in adjunction to systolic function, may be used to guide clinical decision-making in heart failure patients. In contrast, an impaired exercise tolerance suggests an increased risk for death in heart failure in all variations of systolic function, and future clinical trials might therefore emphasize this group of patients in whom treatment effects are likely larger than in patients with normal exercise response.

## 5. Limitations

Mortality data for the current study were derived from the official death registry. While allowing for a near-total follow-up of 99.3%, they do not distinguish all-cause mortality from cardiovascular mortality. In addition, due to the observational nature of the current study and despite using multivariable models adjusting for known confounders, the presence of unknown confounding factors cannot be excluded.

A limitation of the current study is the diagnostic uncertainty of patients who present with clinical signs of heart failure, e.g., exertional dyspnea in the absence of pulmonary causes, yet who have no evidence of systolic dysfunction or elevation of intracardiac pressure at rest or during exercise. For the purpose of the current study, these patients were considered as a control cohort. In addition, due to the retrospective nature of the current study that included patients from 2007 on, systolic function and cardiac chamber sizes were derived from linear measurements, with known limitations in subgroups of patients, particularly those with regional wall motion abnormalities. For five patients (0.7% of the study population), mortality data were unavailable. Despite the large size of the complete study cohort, some subgroups were considerably smaller, which may have potentially affected the prognostic significance of the variables of interest.

## 6. Conclusions

Invasively determined exercise hemodynamics may improve risk stratification of heart failure patients for survival compared to quantification of systolic function alone. Our data suggest that the hemodynamic response to exercise might be valuable for enriching future heart failure randomized controlled trials for patients at higher risk of outcome regardless of LVEF.

## Figures and Tables

**Table 1:** Clinical and echocardiographic characteristics with univariable hazard ratios (HRs) for all-cause mortality.

	All Patients	Survivors	Non-Survivors			
	*n* = 682	*n* = 451	*n* = 231	*p*	HR (95% CI)	*p* (for HR)
General characteristics and comorbidities						
Female Sex	269 (39.2)	182 (40.4)	87 (37.7)	0.157	0.904 (0.69–1.18)	0.460
Age	64.1 (12)	61.1 (11.8)	70.2 (10)	<0.001	1.069 (1.05–1.08)	<0.001
Hypertension	181 (26.3)	148 (32.8)	201 (87)	<0.001	2.621 (1.79–3.85)	<0.001
BMI	27.8 (4.8)	27.5 (4.6)	28.6 (5.0)	0.003	1.033 (1.01–1.06)	0.010
Diabetes	231 (33.6)	65 (14.4)	59 (25.5)	0.001	1.854 (1.38–2.49)	<0.001
Coronary artery disease	282 (41)	154 (34.1)	127 (55)	<0.001	1.992 (1.54–2.58)	<0.001
Atrial fibrillation	180 (26.2)	84 (18.6)	96 (41.6)	<0.001	2.486 (1.91–3.23)	<0.001
NYHA class	2.53 (0.58)	2.50 (0.59)	2.58 (0.57)	<0.05	1.258 (1.006–1.574)	0.044
Serum parameters						
Creatinine (mg/dL)	1.03 (0.4)	0.97 (0.27)	1.15 (0.47)	<0.001	2.352 (1.92–2.88)	<0.001
Hemoglobin (g/dL)	13.9 (1.5)	14.0 (1.4)	13.6 (1.7)	0.001	0.844 (0.78–0.92)	<0.001
Sodium (mmol/L)	140 (3.2)	140 (2.9)	139 (3.7)	0.032	0.961 (0.92–1.00)	0.058
Echocardiography						
Left atrial diameter (mm)	44 (8)	43 (7)	46 (8)	<0.001	1.045 (1.03–1.06)	<0.001
EF_Teichholz_ (%)	59 (16)	60 (16)	58 (17)	0.186	0.995 (0.99–1.00)	0.231
LVEDD (mm)	54 (9)	55 (9)	54 (8)	0.506	0.993 (0.98–1.01)	0.365
LVESD (mm)	37 (10)	37 (10)	37 (10)	0.731	1.000 (0.99–1.01)	0.979
Medication use (*n*)						
Beta-blocker	315 (45.9)	183 (40.6)	131 (56.7)	<0.001	1.519 (1.17–1.97)	0.002
ACE-inhibitors	257 (37.4)	156 (34.6)	101 (43.7)	0.015	1.187 (0.92–1.54)	0.198
AT2-blockers	127 (18.5)	74 (16.4)	51 (22.1)	0.090	1.355 (0.99–1.85)	0.063
Any diuretic	289 (42.1)	154 (34.1)	135 (58.4)	<0.001	2.070 (1.59–2.69)	<0.001

BMI: body mass index, LVEDD: left ventricular end-diastolic diameter, LVESD: left ventricular end-systolic diameter, ACE: angiotensin-converting enzyme, AT1: angiotensin-1 receptor, NYHA: New York Heart Association.

**Table 2:** Resting and exercise hemodynamics in survivors and non-survivors and respective hazard ratios (HRs).

	All Patients	Survivors	Non-Survivors			
	n = 682	n = 451	n = 231	*p*	HR (95% CI)	*p* (for HR)
Peak workload (W)	64.2 (38.3)	72.5 (38.5)	46.3 (30.1)	<0.001	0.980 (0.98–0.99)	<0.001
Peak workload/body weight (W/kg)	0.82 (0.48)	0.91 (0.49)	0.57 (0.35)	<0.001	0.133 (0.13–0.28)	<0.001
Right atrial pressure (mmHg)						
at rest	6.6 (4.03)	6.00 (3.6)	7.9 (4.6)	<0.001	1.073 (1.05–1.10)	<0.001
at peak workload	15.1 (6.6)	13.9 (6.3)	18.0 (6.3)	<0.001	1.069 (1.05–1.09)	<0.001
Pulmonary capillary wedge pressure (mmHg)						
at rest	12.0 (8.0–18.0)	10.0 (8.0–15.0)	14.1 (10.0–21.0)	<0.001	1.049 (1.03–1.07)	<0.001
at peak workload	28.0 (20.0–34.0)	27.0 (20.0–33.0)	29.0 (23.0–35.0)	<0.001	1.025 (1.01–1.04)	0.001
Pulmonary artery pressure (mmHg)						
at rest	23.1 (9.6)	21.2 (8.7)	27.0 (10.5)	<0.001	1.040 (1.03–1.05)	<0.001
at peak workload	43.4 (10.9)	42.1 (10.6)	46.5 (10.6)	<0.001	1.025 (1.01–1.04)	<0.001

**Table 3:** Clinical and echocardiographic characteristics with univariable hazard ratios (HRs) for all-cause mortality.

	Preserved EF (*n* = 512)				Impaired EF (<50%, Includes Both HFmrEF and HFrEF, *n* = 170)			
	Survivors	Non-Survivors			Survivors	Non-Survivors		
	*n* = 342	*n* = 170	HR (95% CI)	*p* (for HR)	*n* = 109	*n* = 61	HR (95% CI)	*p* (for HR)
Female Sex	153 (44.7)	68 (40.0)	0.832 (0.61–1.13)	0.241	29 (26.6)	19 (31.1)	0.867 (0.50–1.49)	0.605
Age	62.2 (11.8)	71.4 (9.8)	1.073 (1.06–1.09)	<0.001	57.5 (11.2)	66.8 (10.0)	1.046 (1.05–1.10)	<0.001
Hypertension	227 (66.4)	148 (87.1)	2.706 (1.73–4.24)	<0.001	76 (69.7)	53 (86.9)	2.255 (1.07–4.74)	0.032
BMI	27.0 (4.4)	28.6 (5.1)	1.050 (1.02–1.08)	<0.001	28.9 (4.8)	28.5 (4.9)	0.980 (0.93–1.04)	0.470
Diabetes	41 (12.0)	43 (25.3)	1.952 (1.38–2.76)	<0.001	24 (22. 0)	16 (26.2)	1.437 (0.81–2.55)	0.214
Coronary artery disease	112 (32.7)	92 (54.1)	1.913 (1.42–2.59)	<0.001	42 (38.5)	35 (57.3)	2.069 (1.24–3.45)	0.005
Atrial fibrillation	64 (18.7)	72 (42.4)	2.497 (1.84–3.39)	<0.001	20 (18.3)	24 (39.3)	2.351 (1.40–3.94)	0.001
Creatine (mg/dL)	0.95 (0.25)	1.13 (0.49)	2.340 (1.85–2.95)	<0.001	1.04 (0.3)	1.20 (0.4)	3.105 (1.70–5.68)	<0.001
Hemoglobin (g/dL)	14.0 (1.3)	13.6 (1.3)	0.848 (0.77–0.94)	0.002	14.3 (1.8)	13.9 (1.5)	0.844 (0.73–0.97)	0.020
Sodium (mmol/L)	140 (3.0)	140 (3.9)	0.976 (0.93–1.02)	0.309	140 (2.6)	139 (3.3)	0.893 (0.82–0.98)	0.015
Left atrial diameter (mm)	43 (7)	45 (9)	1.049 (1.03–1.07)	<0.001	44 (9)	48 (9)	1.036 (1.01–1.07)	0.020
EF_Teichholz_	67 (9)	66 (9)	0.992 (0.98–1.01)	0.396	37 (10)	35 (11)	0.989 (0.96–1.01)	0.372
LVEDD	53 (7)	52 (7)	0.997 (0.98–1.02)	0.788	61 (11)	59 (10)	0.981 (0.96–1.01)	0.130
LVESD	33 (6)	33 (6)	1.003 (0.98–1.03)	0.835	50 (10)	49 (10)	0.986 (0.96–1.01)	0.299

BMI: body mass index, LVEDD: left ventricular end-diastolic diameter, LVESD: left ventricular end-systolic diameter.

## Data Availability

The data presented in this study are available on request from the corresponding author. The data are not publicly available due to privacy.

## Supplementary Materials

The following supporting information can be downloaded at: https://www.mdpi.com/article/10.3390/jcm11195901/s1, Figure S1: Pulmonary capillary wedge pressures during exercise in patients with preserved and impaired left-ventricular ejection fraction.
