# Supplementary material for: Pulmonary Capillary Wedge Pressure during Exercise Is Prognostic for Long-Term Survival in Patients with Symptomatic Heart Failure
Source: J Clin Med. 2022 Oct 6;11(19):5901. doi: 10.3390/jcm11195901 (PMC9570851; doi:10.3390/jcm11195901)
Supplement: Supplementary file 1 [file jcm-11-05901-s001.zip › jcm-1861478-supplementary.pdf]

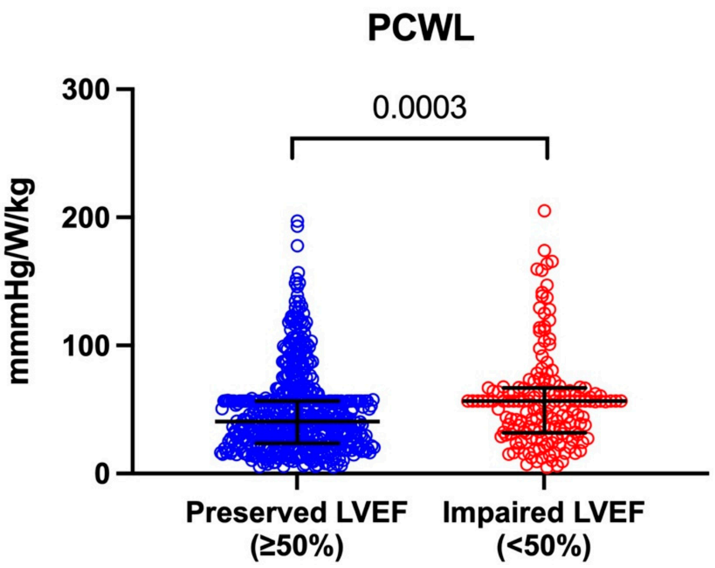

Figure S1: Pulmonary capillary wedge pressures during exercise in patients with preserved and impaired left-ventricular ejection fraction. Pulmonary capillary wedge pressures during exercise, expressed as peak exercise to workload normalized to body weight (PCWL), is higher in patients with impaired left-ventricular ejection fraction <50% (in red) as opposed to patients with preserved systolic function (in blue).
